# A two dimensional electromechanical model of a cardiomyocyte to assess intra-cellular regional mechanical heterogeneities

**DOI:** 10.1371/journal.pone.0182915

**Published:** 2017-08-24

**Authors:** Patricia Garcia-Canadilla, Jose F. Rodriguez, Maria J. Palazzi, Anna Gonzalez-Tendero, Patrick Schönleitner, Vedrana Balicevic, Sven Loncaric, Joost J. F. P. Luiken, Mario Ceresa, Oscar Camara, Gudrun Antoons, Fatima Crispi, Eduard Gratacos, Bart Bijnens

**Affiliations:** 1 Dept. of Information and Communication Technologies, Universitat Pompeu Fabra, Barcelona, Spain; 2 LaBS, Chemistry, materials and chemical engineering department “Giulio Natta”, Politecnico di Milano, Milano, Italy; 3 BCNatal - Barcelona Center for Maternal-Fetal and Neonatal Medicine (Hospital Clínic and Hospital Sant Joan de Deu), Fetal i+D Fetal Medicine Research Center, IDIBAPS, University of Barcelona, Barcelona, Spain; 4 Dept. of Physiology, Maastricht University, Maastricht, The Netherlands; 5 Faculty of Electrical Engineering and Computing, University of Zagreb, Zagreb, Croatia; 6 Molecular Genetics, Maastricht University, Maastricht, The Netherlands; 7 Centre for Biomedical Research on Rare Diseases (CIBER-ER), Madrid, Spain; 8 Institució Catalana de Recerca i Estudis Avançats (ICREA), Barcelona, Spain; LAAS-CNRS, FRANCE

## Abstract

Experimental studies on isolated cardiomyocytes from different animal species and human hearts have demonstrated that there are regional differences in the *Ca*^2+^ release, *Ca*^2+^ decay and sarcomere deformation. Local deformation heterogeneities can occur due to a combination of factors: regional/local differences in *Ca*^2+^ release and/or re-uptake, intra-cellular material properties, sarcomere proteins and distribution of the intracellular organelles. To investigate the possible causes of these heterogeneities, we developed a two-dimensional finite-element electromechanical model of a cardiomyocyte that takes into account the experimentally measured local deformation and cytosolic [*Ca*^2+^] to locally define the different variables of the constitutive equations describing the electro/mechanical behaviour of the cell. Then, the model was individualised to three different rat cardiac cells. The local [*Ca*^2+^] transients were used to define the [*Ca*^2+^]-dependent activation functions. The cell-specific local Young’s moduli were estimated by solving an inverse problem, minimizing the error between the measured and simulated local deformations along the longitudinal axis of the cell. We found that heterogeneities in the deformation during contraction were determined mainly by the local elasticity rather than the local amount of *Ca*^2+^, while in the relaxation phase deformation was mainly influenced by *Ca*^2+^ re-uptake. Our electromechanical model was able to successfully estimate the local elasticity along the longitudinal direction in three different cells. In conclusion, our proposed model seems to be a good approximation to assess the heterogeneous intracellular mechanical properties to help in the understanding of the underlying mechanisms of cardiomyocyte dysfunction.

## Introduction

Cardiomyocytes are the contractile cells that constitute the myocardial wall. They contain myofibrils that, in turn, are composed of long chains of sarcomeres, which are the fundamental cardiac contractile units. Cardiomyocytes can contain one or more nuclei and have a high mitochondrial density [1]. The contraction-relaxation process within a cardiomyocyte is mainly mediated by *Ca*^2+^ [2]. The release of *Ca*^2+^ after electrical activation induces the contraction of the cardiomyocyte (excitation—contraction coupling) [2]. The *Ca*^2+^ influx through sarcolemmal voltage-dependent channels triggers the *Ca*^2+^ release from the sarcoplasmic reticulum (SR) (*Ca*^2+^ induced—*Ca*^2+^ release). This increase in cytosolic *Ca*^2+^ activates the myofilaments that induces cell shortening. To allow cell relaxation, *Ca*^2+^ is extracted from the cytosol, mainly by the *Ca*^2+^ pump (SERCA) into the SR, and by the *Na*^+^/*Ca*^2+^ exchanger (NCX) to the extracellular space [2]. In cardiomyocytes, coordinated *Ca*^2+^ release from the SR determines cellular contraction. *Ca*^2+^-induced *Ca*^2+^ release is regulated locally in subcellular microdomains as demonstrated in some studies providing quantitative evidence for regional differences in the subcellular regulation of *Ca*^2+^ release [3]. Areas of delayed *Ca*^2+^ release were related to regional absence of T-Tubules. Regional differences in *Ca*^2+^ re-uptake during diastole were also reported [4]. In the same study, heterogeneities in the sarcomere relaxation / re-lengthening time were found and, furthermore, the local sarcomere re-lengthening time correlated with the local [*Ca*^2+^] decay time, indicating that dyssynchrony of the decay of cytosolic [*Ca*^2+^] contributes to dyssynchronous intracellular sarcomere re-lengthening. Additionally, some studies have shown that sarcomere relaxation is not uniform in striated muscle [5], and these non-uniform changes are mainly due to regional differences in the rate of *Ca*^2+^ removal [6]. This increased dyssynchrony in intracellular [*Ca*^2+^] decay and sarcomere re-lenghtening may impair cardiomyocyte function [4]. All these findings demonstrate the existence of functional heterogeneities within healthy cardiac cells and also suggest that they may vary and play an important role under pathological conditions, including remodelling and chronic ischemia. For example, the regional loss of T-tubules contributes to contractile dysfunction in chronic ischemia, myocardial hypertrophy and heart failure [3, 7, 8]. Intracellular differences in the arrangement of organelles [9] and in the resting sarcomere length [10] have also been reported in fetal cardiac cells of an animal model of intrauterine growth restriction. Therefore, the study of the intracellular heterogeneities present in cardiac cells is important since it allows us to improve the understanding of the underlying mechanisms of cardiomyocyte dysfunction and to identify biomarkers for the early detection of different cardiomyopathies.

Regional heterogeneities in cell contraction / deformation can be due to several factors: (1) *Ca*^2+^ release and / or re-uptake (timing and amount of *Ca*^2+^) differences, (2) intra-cellular differences in bulk material properties, (3) differences in the local resting sarcomere length, and/or (4) different spatial distribution of the intracellular organelles. In this regard, the spatial distribution of mechanical properties in cardiac cells has been conventionally measured using atomic force microscopy (AFM), demonstrating the existence of regional differences in the elastic modulus [11–15]. However, this technique is tedious, time-consuming and has high measurement variability. For example, the values of the measured Young’s modulus in the same cell type reported in different studies differ from each other [11], making it difficult to know whether the differences are due to the intrinsic heterogeneities in the mechanical properties of the cell or to measurements variability. In addition, AFM determines the “compressive” Young modulus of the bulk and not the elastic modulus along the direction of contraction of the cell. For this reason, finite-element models based on constitutive laws have emerged as an alternative to assess the mechanical properties of the cell and to help in understanding the functional and structural differences between healthy and pathological cells. Several electromechanical models of the heart have been developed during the last years [16–22], which include more or less detailed electrophysiology models, together with the modelling of the passive and active mechanical tissue properties. However, our aim is to study the mechanical heterogeneities within a cardiac cell and therefore a specific model of a single cardiomyocyte is needed. A number of two and three-dimensional models of cardiomyocytes have been published in the last two decades [23–25], all based on the continuum mechanic framework in which the mechanical behaviour is described by strain energy functions. However, in all published models, a homogeneous distribution of cell mechanical properties has been assumed, whereas experimental studies in living cardiomyocytes have demonstrated that regional heterogeneities exist. Moreover, all these models were not individualised to patient-specific data. Inverse problems for patient-specific modelling are becoming more popular for obtaining information about physical properties that cannot be otherwise measured [26, 27].

The aim of this study is to develop a simplified two-dimensional electromechanical cell model that takes into account the experimentally measured local deformation and cytosolic [*Ca*^2+^] to locally determine the different parameters of the constitutive equations. Hence, the resulting finite element electromechanical model of a cardiomyocyte accounts for the heterogeneities in the mechanical properties of the cell. We assessed the relationship between cell contraction and regional mechanical properties such as elasticity, and the amount of active force generated by the sarcomeres and cytosolic [*Ca*^2+^], in an effort to explain the observed local differences in the amount and duration of cell contraction. Local cytosolic [*Ca*^2+^] transients and local deformation along the longitudinal direction of the cell were measured by confocal line-scan microscopy, in three electrically stimulated rat cardiac cells. This data was used to individualise the model and to estimate the local Young modulus along the longitudinal axis of the cell by means of an inverse finite element problem, which minimises the error between the experimentally measured local deformation and the one obtained from numerical simulations.

## Materials and methods

### Ethical approval

All procedures were approved by the Animal Care and Use Committee of Maastricht University (DEC2012-073) and complied with European legislation on animal experimentation (Directive 2010/63/EU).

### Experimental protocol

#### Cardiomyocyte imaging

Left ventricular myocytes were obtained from three male Lewis rats (∼250g, Charles Rivers, Netherlands), fed ad libitum and housed at 21°C with a 12h light-dark cycle, following an isolation procedure described previously [28]. In short, animals were anesthetized with sodium pentobarbital (150–200 mg/kg i.p.) and then hearts were quickly excised, mounted on a Langendorff apparatus and retrogradely perfused with collagenase/protease solution.

Real-time imaging of *Ca*^2+^ and T-tubule membrane deformation was performed with confocal line-scan microscopy (Zeiss, LSM 700, Jena, Germany). For doing so, cells were dual-loaded with the *Ca*^2+^ dye Fluo-4 AM (3 M, for 10 min, followed by 15 min de-esterification) and the membrane-selective fluorescence dye FM4-64 (5 M for 10 min; Life technologies/Invitrogen). A scan line was selected parallel to the longitudinal axis, extending across the full length of the cardiomyocyte in the focal plane and orthogonal to the Z-lines of the sarcomeres. Fluorescence was excited at 488 nm; the emitted light was separated by a variable secondary dichroic set at 590 nm for simultaneous detection of Fluo-4 (peak emission at 512 nm) and FM4-64 (emission >640 nm). Light images were collected by transmitted light detection. In superimposed line-scans, the T-tubule signal appeared as a regular spaced pattern marking the Z-lines of the sarcomeres, and was taken as a measure to image local sarcomere movement in the confocal plane over time.

**Fig 1:**
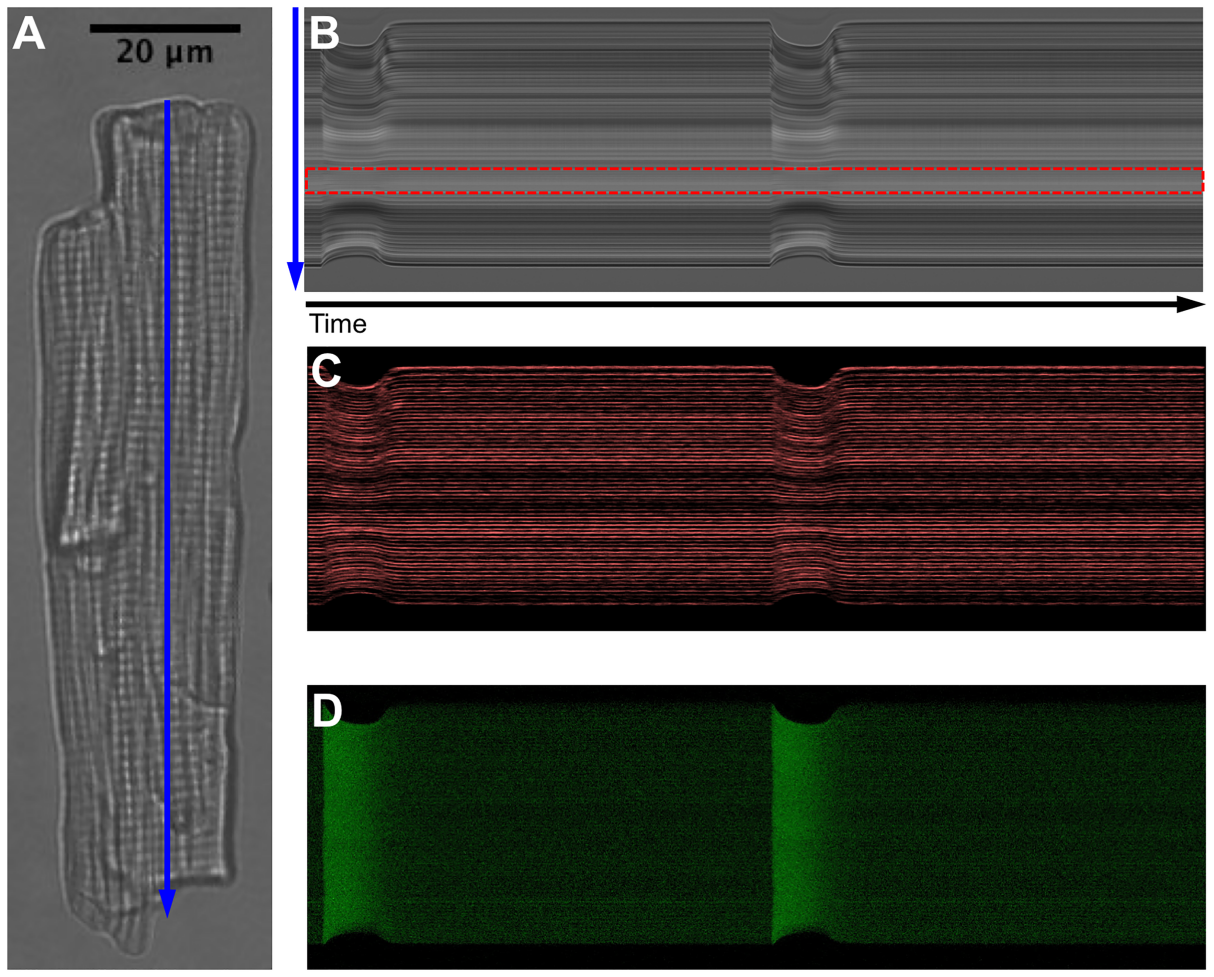
Different images recorded during the cardiomyocyte electrical stimulation experiments, with a pacing rate of 1Hz. A: Transmitted light image of the whole cell. The blue arrow corresponds to the line-scan where the images acquisition was performed. B: Line-scan transmitted light image. The red box indicates a region within the cell with zero displacement. C: Confocal FM4-64 image where the T-Tubule and sarcolemma are visible. D: Confocal Fluo-4 image corresponding to cytosolic [*Ca*^2+^]. The vertical axis corresponds to the line-scan (blue arrow) and the horizontal one to the time. The line-scan images resolution is 3.2 ⋅ 10^−3^ *s* × 0.28*μm*.

The experimental superfusate was a Tyrode solution containing (in mmol/L) NaCl 136, KCl 5.4, CaCl2 1.8, MgCl2 1, HEPES 10, and glucose 10; pH was adjusted to 7.40 with NaOH. Cells were electrically stimulated at 1Hz until steady state cytosolic calcium transient was reached. Line-scan images of 5 to 10 successive beats were recorded. An example can be seen in Fig 1. All experiments were performed at 37°C.

#### Measures of local sarcomere strain

The distance between adjacent sarcomeres (local sarcomere length) was manually measured in FM4-64 line-scan images, at 15 spaced time points between the initiation of local contraction and the maximal local re-lengthening (end of the beat), always at the time of maximal local contraction, as performed in Hohendanner et al. [4]. Therefore, the local strain profile for each sarcomere within the line-scan was obtained by data interpolation of the 15 time points. These measurements were performed for all the sarcomeres along the line-scan, approximately between 30–50. Then, local strain curves from the different sarcomeres were averaged and the time instant corresponding to the maximum cellular contraction was identified. Finally, a curve of the local strain at maximum contraction along the line-scan was obtained.

#### Measures of local [*Ca*^2+^] transients

The local [*Ca*^2+^] transients (*F*) were obtained for all points of the line-scan where local strain was manually measured, from the [*Ca*^2+^]-dependent fluorescence intensity line-scan images (Fig 1D), using the variation in pixel position across time due to cell contraction. The global [*Ca*^2+^] transient was then obtained as the average of all local transients. Global and local [*Ca*^2+^] transients were normalised to the fluorescence intensity at rest (*F*_0_).

### Description of the model

#### Passive mechanical properties of the cardiomyocyte

Usually, cardiac tissue is considered a orthotropic or transversaly isotropic hyperelastic material. However, when modelling a single cardiac cell, the passive mechanical behaviour of the cellular medium is often modelled as an isotropic hyperelastic material [23–25, 29, 30], while the anisotropy is introduced through the active (contractile) behaviour of the cell. In this work, similarly to previous cardiomyocyte models [24], the cell was assumed to behave as a nearly-incompressible hyperelastic Mooney-Rivlin medium, characterised by a strain-energy function given by:

$$\begin{array}{c} W=C_{10}\left({\bar{I}}_{1}-3\right)+C_{01}\left({\bar{I}}_{2}-3\right)+D_{1}{\left(J-1\right)}^{2} \end{array}$$ (1)

where ${\bar{I}}_{1}$ and ${\bar{I}}_{2}$ are the first and second invariants of the modified right Cauchy-Green strain tensor, $\bar{C}=J^{-2/3}C$, with *J* the local volume change. *C*_10_, *C*_01_ and *D*_1_ are material constants, with *μ* = 2(*C*_10_ + *C*_01_) the initial shear modulus. *C*_01_ was chosen as *C*_10_/50. The material constants can be related to the initial elastic Young’s modulus, (*E*), and the initial Poisson’s ratio (*ν*) through the following relationships: *E* = 9*Kμ*/(3*K* + *μ*) and *ν* = (3*K* − 2*μ*)/2(3*K* + *μ*), where the Poisson’s ratio was set to 0.49 and *D*_1_ defined as half the finite bulk modulus, *K* = 2*D*_1_, since we assume a nearly incompressible material. A more detailed description of the model is given in S1 Appendix. Differently to previous studies [23–25, 29, 30] the material parameters of the strain energy function are not assumed constant throughout the length of the cell (direction of cell’s contraction) but they are allowed to vary, i.e., the model accounts for heterogeneity in the mechanical properties of the cell in the longitudinal direction and assumes them to be uniform in the cross section of the cell.

#### Active mechanical properties of the cardiomyocyte

The active stress generated by the active fibres within the cell was modelled as an active tension, *T*_*act*_, generated along the direction given by the sarcomere orientation in the reference configuration, **d**^**k**^, calculated as: **S**_*act*_ = *T*_*act*_ **D**^*k*^, where the tensor **D**^*k*^ = **d**^*k*^ ⊗ **d**^*k*^ is defined by the fibre system direction vector **d**^*k*^. Hence, an anisotropic active contraction of the cardiomyocyte is assumed. In this regard, only fibres in the longitudinal direction were considered so *k* = 1. The active tension was computed as [29, 31, 32]:

$$\begin{array}{c} T_{act}=A\cdot f_{max}\cdot exp\left\{-{\left(\frac{\varepsilon -\varepsilon_{opt}}{s}\right)}^{2}\right\} \end{array}$$ (2)

where $\varepsilon =\frac{1}{2}\left(\bar{C}:D^{k}-1\right)$ is the Green strain along the fibre direction **d**^*k*^, *ε*_*opt*_ is the optimal deformation at the maximal activation state, and *s* represents the sensitivity to the actin-myosin overlap. The parameter *f*_*max*_ is the maximal tension that can be delivered by the sarcomere. *A* is a [*Ca*^2+^]-dependent function modelling the excitation-contraction coupling. Parameters *ε*_*opt*_, *s* and *f*_*max*_ were identified from experimental data acquired on skinned rat cardiac myocytes [33] by means of a nonlinear regression procedure as in Tracqui et al. [29]. The overall set of data points reported by Weiwad et al. [33] was fitted for pCa (pCa = −*log*[*Ca*^2+^]) ranging from 4.9 to 5.7 with the expression of active stress *T*_*act*_ (Eq 2), for a resting sarcomere length *SL*_0_ = 1.9*μm* and a Hill exponent *nH* = 2.6, in agreement with the experimental data [33]. The values identified after the non-linear regression procedure were *f*_*max*_ = 54.33, *ε*_*opt*_ = 0.23 and *s* = 0.24 (see S1 Fig).

It is known from experimental data [33] that the amount of active stress that can be generated by the sarcomeres (*T*_*act*_) depends on the amount of cytosolic *Ca*^2+^ available. Therefore, *A* was defined as an activation function modelling the [*Ca*^2+^]-dependent contraction process. This process was described as follows:

$$\begin{array}{c} A\left(t\right)=\frac{Z_{max}^{nH}}{Z_{50}^{nH}+Z_{max}^{nH}}\cdot Z\left(t\right)/Z_{max}\cdot f_{TCa}\left(t\right) \end{array}$$ (3)

The first term of the equation correspond to a Hill-type sigmoid function [29], where *nH* is the Hill coefficient (*nH* > 0.0), *Z*_*max*_ represents the maximum peak of cytosolic [*Ca*^2+^] and *Z*_50_ the half-maximum concentration of cytosolic [*Ca*^2+^]. The second term, *Z*(*t*)/*Z*_*max*_, corresponds to the normalised [*Ca*^2+^] transient. *Z*(*t*) was approximated as a sum of two exponential functions as follows:

$$\begin{array}{c} Z\left(t\right)=a\left(e^{-k_{fall}\cdot t}-e^{-k_{rise}\cdot t}\right)+b \end{array}$$ (4)

where *a* and *b* define the amplitude and baseline of the [*Ca*^2+^] transient and *k*_*fall*_ and *k*_*rise*_ the rate constants of [*Ca*^2+^] decay and rise respectively. Therefore, the corresponding time constants were calculated as *τ*_*fall*_ = 1/*k*_*fall*_ and *τ*_*rise*_ = 1/*k*_*rise*_. For our simulations, the Hill coefficient *nH* was set to 2.6 following [33], and *Z*_50_ was calculated from the global [*Ca*^2+^] transient. The last term in Eq 3, *f*_*TCa*_(*t*), is a unitary function that describes the temporal behaviour of the [*Ca*^2+^] binding to and dissociation from troponin (*TnC*) in the myofilaments, and therefore the [*Ca*^2+^] regulation of contraction [34]. It was defined as the multiplication of two exponentials as follows:

$$\begin{array}{c} f_{TCa}\left(t\right)=\frac{\left(1-e^{-{\left(t/\tau_{c}\right)}^{\beta }}\right)\left(e^{-{\left(\left(t-t_{b}\right)/\tau_{r}\right)}^{\beta }}\right)}{\left(1-e^{-{\left(t_{p}/\tau_{c}\right)}^{\beta }}\right)\left(e^{-{\left(\left(t_{p}-t_{b}\right)/\tau_{r}\right)}^{\beta }}\right)} \end{array}$$ (5)

where *τ*_*c*_ and *τ*_*r*_ are the contraction and relaxation time constants respectively, *β* is an exponent between 1 and 2, and *t*_*p*_ corresponds to the time to peak, such that maximum of *f*_*TCa*_ is 1. Due to the lack of experimental force measurements, these parameters were identified by means of a non-linear regression consisting on minimising the error between the simulated and experimentally measured normalised global cell deformation. However, if the force developed by the cell during contraction was available, *f*_*TCa*_ could be fitted to the force measurement and therefore, *f*_*TCa*_ will account for the tension dependence *Ca*^2+^ binding to *TnC*.

**Fig 2:**
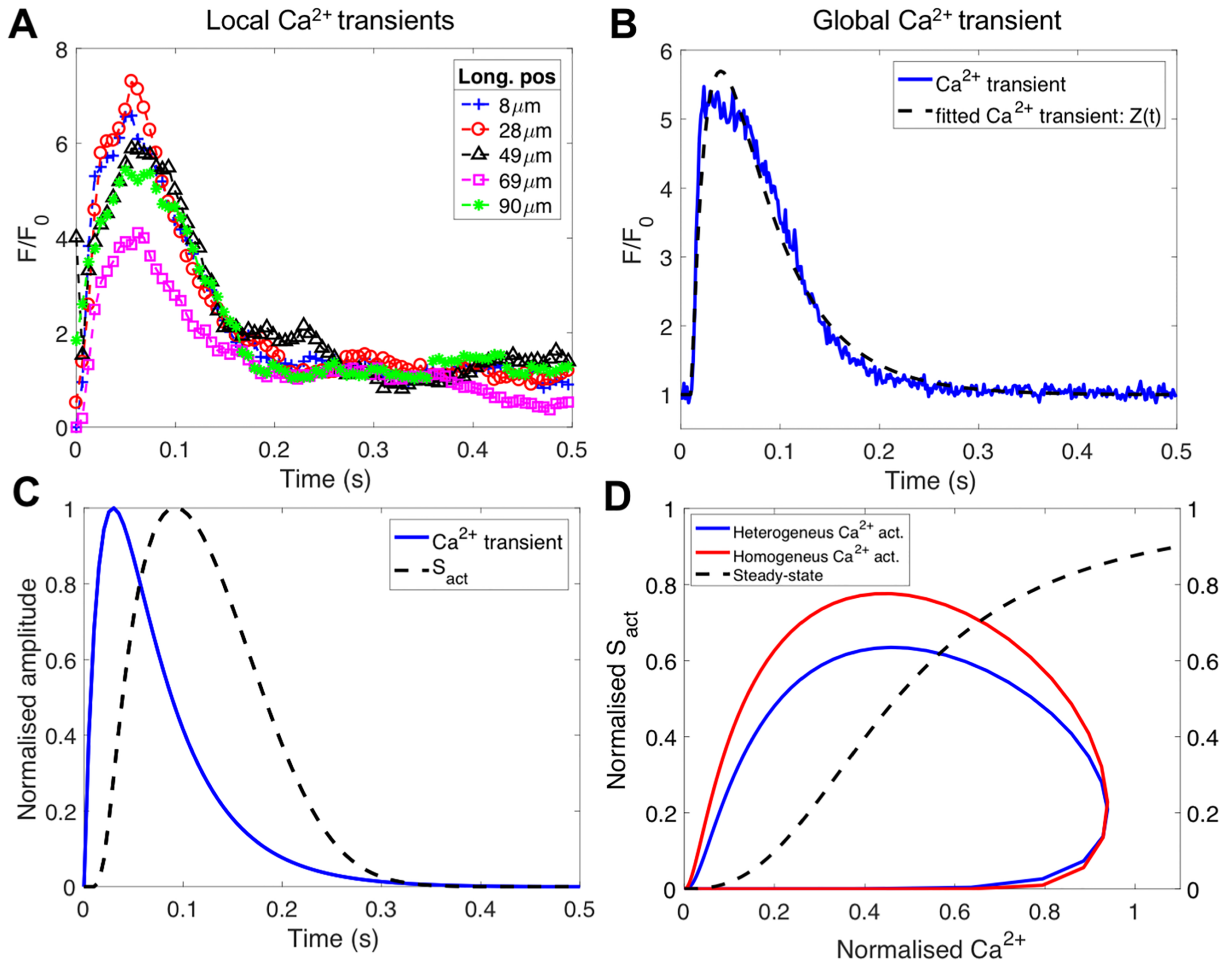
Experimental measured local and global [*Ca*^2+^] transients and dynamic vs. steady-state force and [*Ca*^2+^] relationship. A: Experimental measured local [*Ca*^2+^] transients normalised to basal fluorescence (*F*_0_) at different positions along the longitudinal axis of the cell (Long. pos). B: Global experimental measured (solid line) and fitted (dash line) with the two exponential functions (*Z*(*t*) in Eq 4) [*Ca*^2+^] transients. C: Individual time course of cytosolic [*Ca*^2+^] and active stress (*S*_*act*_). D: Phase-plane plot relating force to *Ca*^2+^ for both heterogeneous and homogeneous *Ca*^2+^ activation. The dynamic behaviour for a single contraction is compared with the steady-state relation.

In order to account for the longitudinal [*Ca*^2+^] heterogeneity, *A*(*t*) was defined locally at each point of the line-scan. Local and global [*Ca*^2+^] transients were obtained from experimental data. Fig 2A and 2B show the experimentally measured local and global [*Ca*^2+^] transients respectively and Fig 2B shows also the function *Z*(*t*) fitted to the global [*Ca*^2+^] transient. Note that using the actual [*Ca*^2+^] transients measured in the cell, makes our simulation independent of the stretch-sensitive channels present in the cell membrane, that will otherwise be important in a fully electro-mechanical analysis of the cell contraction. Fig 2C and 2D show the time course of force and *Ca*^2+^ transients and the dynamic force-*Ca*^2+^ phase-plane plots for a single contraction alongside the steady state plot respectively, showing a good agreement with what was experimentally measured in rat left ventricular cells [35, 36].

### Finite element simulation of a cardiomyocyte contraction

#### Numerical equations and boundary conditions

A two dimensional in plane-stress is considered to model the active contraction of the cell. The model is justified by the fact that the thickness to length ratio of the cell is in the order of 1:10. In this case, it is also assumed that the out-of-plane deformation is responsible for keeping the volume of the cardiomycyte constant. The equilibrium equation is defined as [31, 32]:

$$\begin{array}{c} \int_{\Omega^{\left(0\right)}}\left(S^{eff}\left(u\right)+K\left(J-1\right)JC^{-1}\right):d\varepsilon \left(v\right)dV=0,\forall v \end{array}$$ (6)

where **S**^*eff*^(**u**) is the effective stress calculated as the sum of passive (**S**_*pas*_) and active (**S**_*act*_) stress **S**^*eff*^ = **S**_*pas*_ + **S**_*act*_, *ε* is the Green Strain tensor, Ω^(0)^ is the initial (undeformed) configuration, **u** is the displacement vector and **v** represents a test function of the weak formulation. **S**_*pas*_ was calculated as [37]:

$$\begin{array}{c} S_{pas}\left(u\right)=2C_{10}J^{-\frac{2}{3}}\left(I-\frac{1}{3}\mathrm{tr}\left(C\right)C^{-1}\right)+2C_{01}J^{-\frac{4}{3}}\left(\mathrm{tr}\left(C\right)I-C-\frac{2}{6}\left({\left(\mathrm{tr}C\right)}^{2}-\mathrm{tr}\left(C^{2}\right)\right)C^{-1}\right) \end{array}$$ (7)

and **S**_*act*_ = *T*_*act*_ **D**^*k*^ where *T*_*act*_ was calculated as described in Eq 2. The equilibrium equation (Eq 6) was numerically solved using the finite element method with the software SfePy [37] in the 2D domain. A detailed mathematical description of the model equations together with the solver’s parameters used for solving the finite element problem are given in S1 Appendix.

The Robin boundary condition, **S** ⋅ **n** + *α* ⋅ **u** = 0, was imposed on the cell boundary Γ_0_, where **n** is the normal vector to cell membrane Γ_0_ and *α* is a parameter representing the elastic response due to the presence of surrounding fluid hindering and constraining the motion of the cell. Parameter *α* was set initially to *α* = 1 ⋅ 10^−2^ kPa, following Ruiz-Baier et al [24] which demonstrated that this kind of boundary condition seems to be the more suitable for modelling free cardiac cells since it represents the elastic behaviour of the surrounding tissue, contact with other myocytes, or fluid hindering and constraining the motion of the cell, being more biophysically tunable and mimicking better the real cell-matrix and cell-cell adhesions of myocytes. In addition, a Dirichlet boundary condition (zero displacement condition) was imposed on the cell region with zero or positive strain, since it corresponds to an observed region with zero displacement (see the red box in Fig 1B, which are believed to correspond to local adhesion sites of the cell or the location of the nucleus of the cell.

#### Estimation of the local elasticity from deformation

The local elastic Young’s moduli (*E*_*i*_) along the longitudinal axis of the cell were estimated automatically by a constrained non-linear optimisation algorithm implemented in MATLAB. It consists of minimising an objective function, *J*, defined as the root mean square error (RMSE) between the simulated and original experimental strain profiles along the longitudinal axis of the cell at the maximum contraction time frame. Hence, the estimation problem consists of searching the parameter set, *θ*, that minimises **Φ** starting from an initial parameter set ($\theta^{0}=E_{i}^{0}$), until the stopping criterion ‖*θ*^*n*^ − *θ*^*n*+1^‖ < 10^−6^ on **Φ** is reached. In order to avoid local minimum solutions, we repeated the procedure several times with different *θ*^0^ keeping the parameter set with a minimum value of **Φ**. However, the estimation of the elasticity for each element along the longitudinal axis of the mesh implies that the number of variables to estimate is proportional to the number of elements in the mesh thus unacceptably increasing the computational burden. Therefore, we propose to estimate the local *E*_*i*_ in two steps. First, a less refined estimation was performed by diving the cell in approximately 20 regions along the cell’s longitudinal axis, and assuming a piecewise linear variation of the logarithm of the elastic Young’s modulus along the longitudinal axis of the cell as:

$$\begin{array}{c} log\left(E_{i}\left(x\right)\right)=\frac{log\left(E_{i+1}^{0}\right)-log\left(E_{i}^{0}\right)}{x_{i+1}-x_{i}}\left(x-x_{i}\right)+log\left(E_{i}^{0}\right),\;x_{i}\leq x\leq x_{i+1} \end{array}$$ (8)

where *x*_*i*_ and *x*_*i*+1_ define the beginning and end of the region *i*, and $E_{i}^{0}$ and $E_{i+1}^{0}$ the estimated Young modulus corresponding to these positions.

After the first minimisation process is completed, a second optimisation step is performed in which, the cardiomyocyte was divided in approximately 200 segments along the longitudinal direction, where the local Young’s modulus was determined. The Young moduli identified in the first step were interpolated into the new segments and used as initial seeds for the second optimisation algorithm.

#### Sensitivity analysis of the local strain

A sensitivity analysis of the main model parameters on the contractile properties predicted by the electromechanical model was performed. The input variables were: (1) [*Ca*^2+^] transient amplitude (*Z*_*max*_), (2) time constant of [*Ca*^2+^] transient decay (*τ*_*fall*_), (3) Young’s Modulus (*E*), (4) *f*_*max*_, (5) *ε*_*opt*_ and (6) *s*. The output variables were: (1) maximum contraction in the longitudinal direction ($\varepsilon_{l}^{max}$), (2) time constant of sarcomere re-lengthening (*τ*_*sl*_) and (3) maximum strain rate in the longitudinal direction (${\dot{\varepsilon }}_{l}^{max}$).

The nominal values of the model parameters were: *Z*_*max*_ = 10.79, *τ*_*fall*_ = 0.08*s*, *f*_*max*_ = 54.33, *ε*_*opt*_ = 0.23 and *s* = 0.24. A 10% variation from the nominal values of *Z*_*max*_, *τ*_*fall*_, *f*_*max*_, *ε*_*opt*_ and *s* was considered. In the case of *E*, it was varied from 10 to 100 kPa. A Montecarlo simulation with 625 sample points was used for the sensitivity analysis. These combinations of model parameters were used with the two dimensional finite element model to generate a set of simulation results from where the contractile properties previously described were determined. A multivariate linear regression analysis was performed to evaluate the relationship between parameters and outputs of the model.

### Validation of the inverse problem procedure

The proposed inverse finite element framework was validated in-silico using synthetic data. A 2D mesh of a synthetic cell of dimensions 117*μm* × 32*μm* with 7742 triangular elements and 4009 nodes, mimicking the real shape of an adult rat cardiomyocyte was constructed with Gmsh [38]. Then, the Young’s modulus of the synthetic cell was randomly defined in 30 different points along the longitudinal axis of the cell within the range of [0.1, 100]kPa. The *E* value of the remaining points was interpolated using a cubic B-spline. The average *E* (mean ± std) within the synthetic cell was 36 ± 11 kPa, which is consistent with previous estimated Young’s modulus in cardiac cells [25]. The synthetic local [*Ca*^2+^] transients were defined by randomly varying the amplitude (*a* and *b* parameters in Eq 4) and the decay time (*τ*_*fall*_ = 1/*k*_*fall*_ parameter in Eq 4). The average amplitude, $Ca_{max}^{2+}$, and *τ*_*fall*_ of the [*Ca*^2+^] transients were 10 ± 1 arb. unit and 0.25 ± 0.03 s respectively.

A single contraction of the cell was simulated by solving the forward electromechanical model (Eq 6) using Sfepy [37] in the two-dimensional meshed domain given the original (synthetic) parameter set (*E*_0_). Then, observations were obtained using the simulated longitudinal strain at maximum contraction time frame, and considered as the experimental strain for the inverse problem. Subsequently, starting from a given parameter set *E*, different from the one used for the forward simulation, the inverse problem described previously was solved to recover the original (synthetic) local Young’s modulus. The robustness of the method in the presence of imprecise measurements was evaluated by adding (0, 5, 10 and 15)% of additive white gaussian noise to the local deformation issued from the forward problem, as done in similar in-silico procedures [39, 40].

### Cell-specific simulation of a cardiomyocyte contraction

#### Defining cell-specific input data of the model

The model was individualised to three different rat cardiomyocytes. To do this, the transmitted light images from the cardiac cells (see Fig 1A) were manually delineated to obtain the cell boundary. Then, 2D meshes with triangular elements were built with Gmsh [38].

The activation function *A*(*t*) was defined using Eq 3, where *Z*(*t*) was obtained from fitting the experimentally measured normalised [*Ca*^2+^] transients (*F*/*F*_0_) to Eq 4.

Two possible scenarios were considered. First, a homogeneous [*Ca*^2+^]-dependent activation in the whole cell (calculated from the experimentally measured global [*Ca*^2+^] transient) was assumed. The second scenario considers the different local [*Ca*^2+^]-dependent activation functions, calculated from the experimentally measured local [*Ca*^2+^] transients.

#### Inverse problem strategy to estimate local cell elasticity

Local elastic Young’s moduli (*E*_*i*_) along the line-scan were estimated following the two-step inverse problem procedure described previously. The parameters *f*_*max*_ and *α* from the active stress function and Robin boundary condition respectively, were also included in the minimisation algorithm in order to improve the inverse-problem results, since they can also have an influence on the global behaviour of the cell. Then, the mean Young’s modulus ($\bar{E}$), the global cell shortening and the Pearson’s correlation between *E*_*i*_ and maximum local longitudinal strain were computed for both simulated scenarios.

## Results

### Sensitivity analysis of the local strain

The results of the multivariate linear regression analysis between model parameters and contractile properties are shown in Table 1. The maximum contraction depends not only on the amount of available [*Ca*^2+^] and on the sensitivity of the actin-myosin overlaps of the sarcomeres but also on the passive elasticity of the cell. In fact, the maximum correlation was obtained for the passive elasticity of the cell. On the contrary, ${\dot{\varepsilon }}_{l}^{max}$ was equally determined by the amplitude of the [*Ca*^2+^] transients and cell elasticity properties. Finally, the time for relaxation mainly depends on how fast the [*Ca*^2+^] is re-uptaken and/or extracted from the cytosol.

**Table 1:** Coefficients of the multivariate linear regression analysis for the dependent variables: Maximum contraction amplitude ($\varepsilon_{l}^{max}$), time of re-lengthening (*τ*_*sl*_) and maximum strain rate (${\dot{\varepsilon }}_{l}^{max}$). The independent variables included in the analysis were: $Ca_{max}^{2+}$, [*Ca*^2+^] transient amplitude; *τ*_*fall*_, time constant of [*Ca*^2+^] transient decay; *E*, Young’s Modulus; *f*_*max*_, the maximal tension delivered by the sarcomere; *ε*_*opt*_, the optimal deformation at the maximal activation state; *s*, the sensitivity to the actin-myosin overlap.

Variables	$\varepsilon_{l}^{max}$	*τ*_*sl*_	${\dot{\varepsilon }}_{l}^{max}$
$Ca_{max}^{2+}$	−0.303**	0.278**	−0.596**
*τ*_*fall*_	0.009	0.905**	0.226**
*E*	0.727**	−0.119**	0.526**
*f*_*max*_	−0.095**	0.017	−0.064**
*ε*_*opt*_	0.254**	-0.001	0.202**
*s*	−0.359**	0.009	−0.267**

** *p* < 0.001.

### Validation of the inverse problem procedure

**Fig 3:**
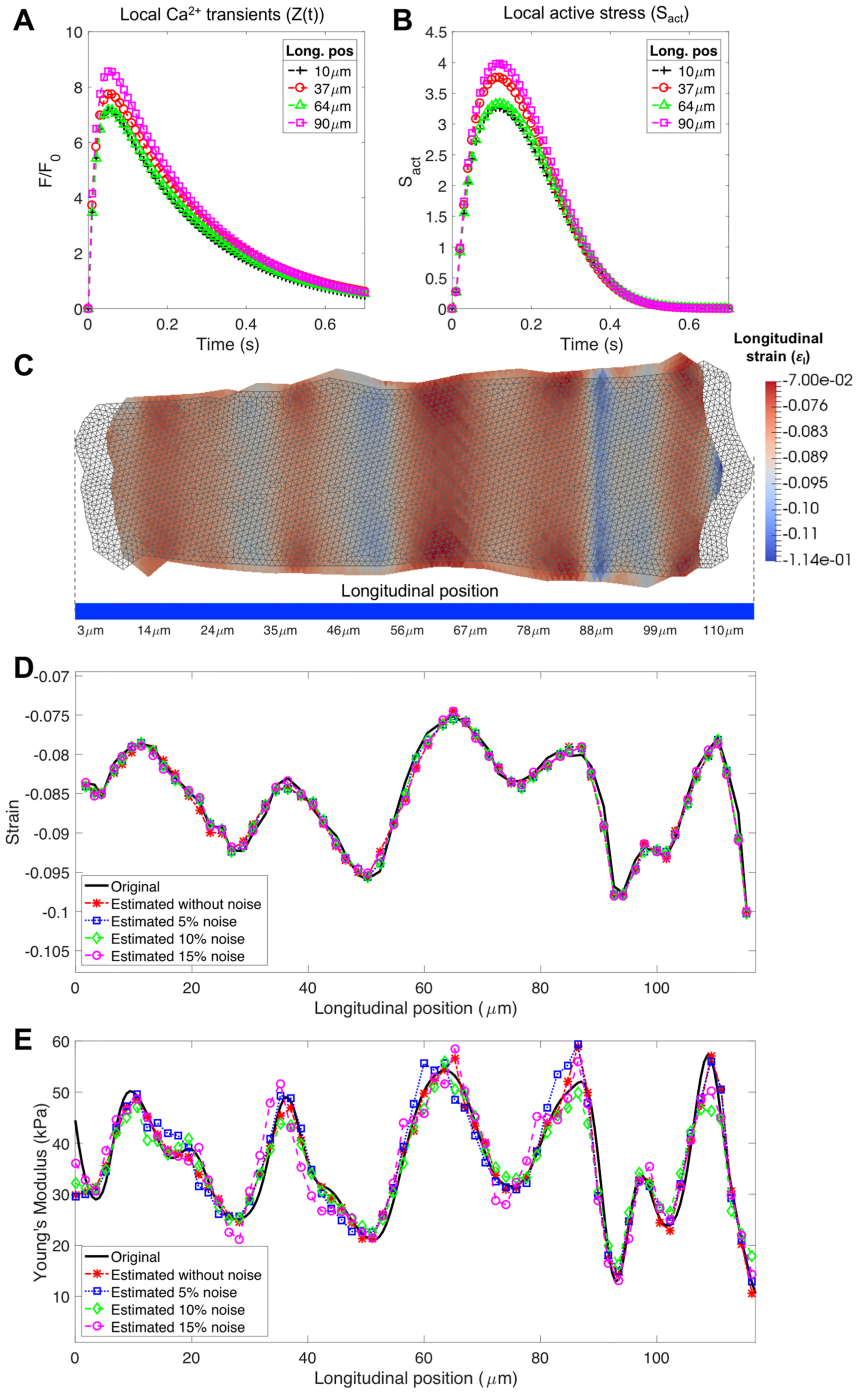
Synthetic data generated for validating the inverse problem procedure and results of the proposed framework validation in presence of gaussian noise. A: Local [*Ca*^2+^] transients. B: Active stress *S*_*act*_(*t*) at different longitudinal positions (Long. pos) of the synthetic cell. C: Undeformed (grey) and deformed mesh of the synthetic cell at maximum contraction time frame. Colormap indicates the simulated longitudinal strain. D: Original (black solid line) and simulated strains along the longitudinal axis of the cell at maximum contraction time frame after the optimisation process with 0% (*), 5% (□), 10% (◇) and 15% (∘) of noise. E: Original (black solid line) and estimated local Young’s moduli along the longitudinal axis (line-scan) of the cell with 0% (*), 5% (□), 10% (◇) and 15% (∘) of noise.

**Fig 4:**
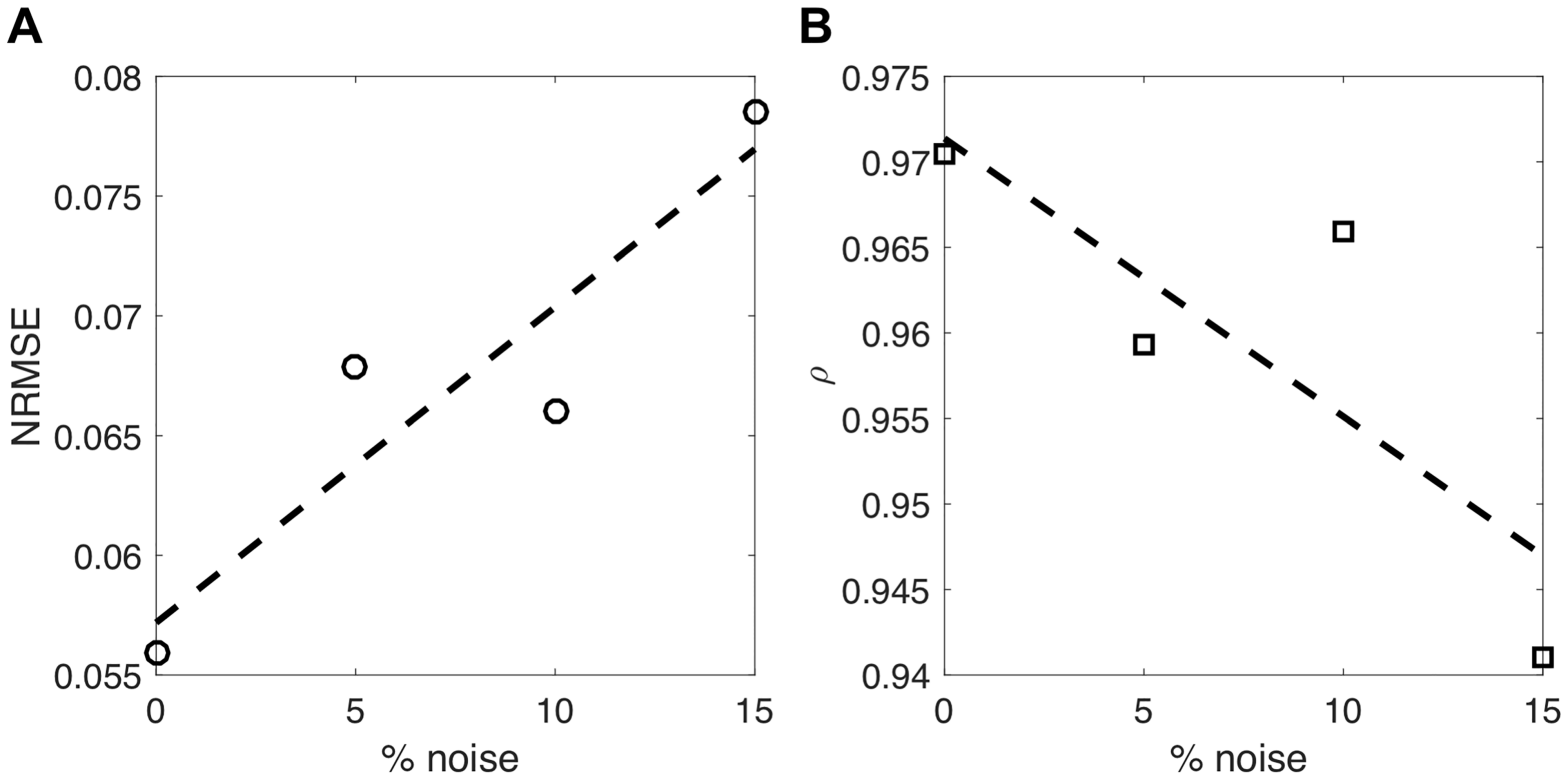
Agreement evaluation between original and estimated local Young’s moduli. A: Normalised root mean square error (NRMSE). B: Pearson’s correlation (*ρ*) between original and estimated local Young’s moduli.

Fig 3 shows the generated local [*Ca*^2+^] transients (Fig 3A) and their corresponding active stresses *S*_*act*_(*t*) (Fig 3B) and the 2D undeformed (non-coloured grid) and deformed (colour-coded with the longitudinal strain) meshes of the synthetic cell, at maximum contraction time (Fig 3C); The comparison between the original (experimental) ($\varepsilon_{l}^{max}$) and the simulated (${\hat{\varepsilon_{l}}}^{max}$) strain profiles along the longitudinal axis of the cell with different noise levels after the minimization process is depicted in Fig 3D. It shows a very good agreement with a RMSE between $\varepsilon_{l}^{max}$ and ${\hat{\varepsilon_{l}}}^{max}$ after the minimisation process below 0.001 (less than 1% error) for all the noisy scenarios. Actual and estimated Young’s moduli along the longitudinal axis of the cell are plotted in Fig 3E, showing also good agreement. To reinforce these results, the normalised RMSE (NRMSE) between the actual and estimated Young’s moduli (Fig 4A) and Pearson’s correlation coefficient (Fig 4B) were plotted to show that even with a 15% of added noise, the NRMSE was below 0.1 and the *ρ* coefficient was above 0.90.

### Cell-specific simulation of a cardiomyocyte contraction

Fig 1 shows different images recorded during the cardiomyocyte electrical stimulation experiments for one of the cells. The cell morphology and the line-scan (in blue) along the longitudinal direction of the cell can be seen in Fig 1A. On the right panel of Fig 1, the three line-scan images are plotted for two consecutive beats: the transmitted light image where the sarcomeres are visualised (Fig 1B) the confocal FM4-64 image corresponding to the sarcolemma and T-tubule network (Fig 1C) and the confocal Fluo-4 image corresponding to cytosolic [*Ca*^2+^] (Fig 1D). The vertical axis corresponds to the line-scan (blue arrow) and the horizontal axis to time. Note that a region within the cell with zero displacement (red rectangle in Fig 1B) can be identified.

Local and global [*Ca*^2+^] transients corresponding to the cell illustrated in Fig 1 are depicted in Fig 2. A large variability in the local [*Ca*^2+^] transients, denoted by the observed differences in the peak amplitude, can be observed in Fig 2A. The approximation of the global [*Ca*^2+^] transient with the two exponential functions is shown in Fig 2B, the excellent fitting provided by the approximation is clearly noticed.

**Fig 5:**
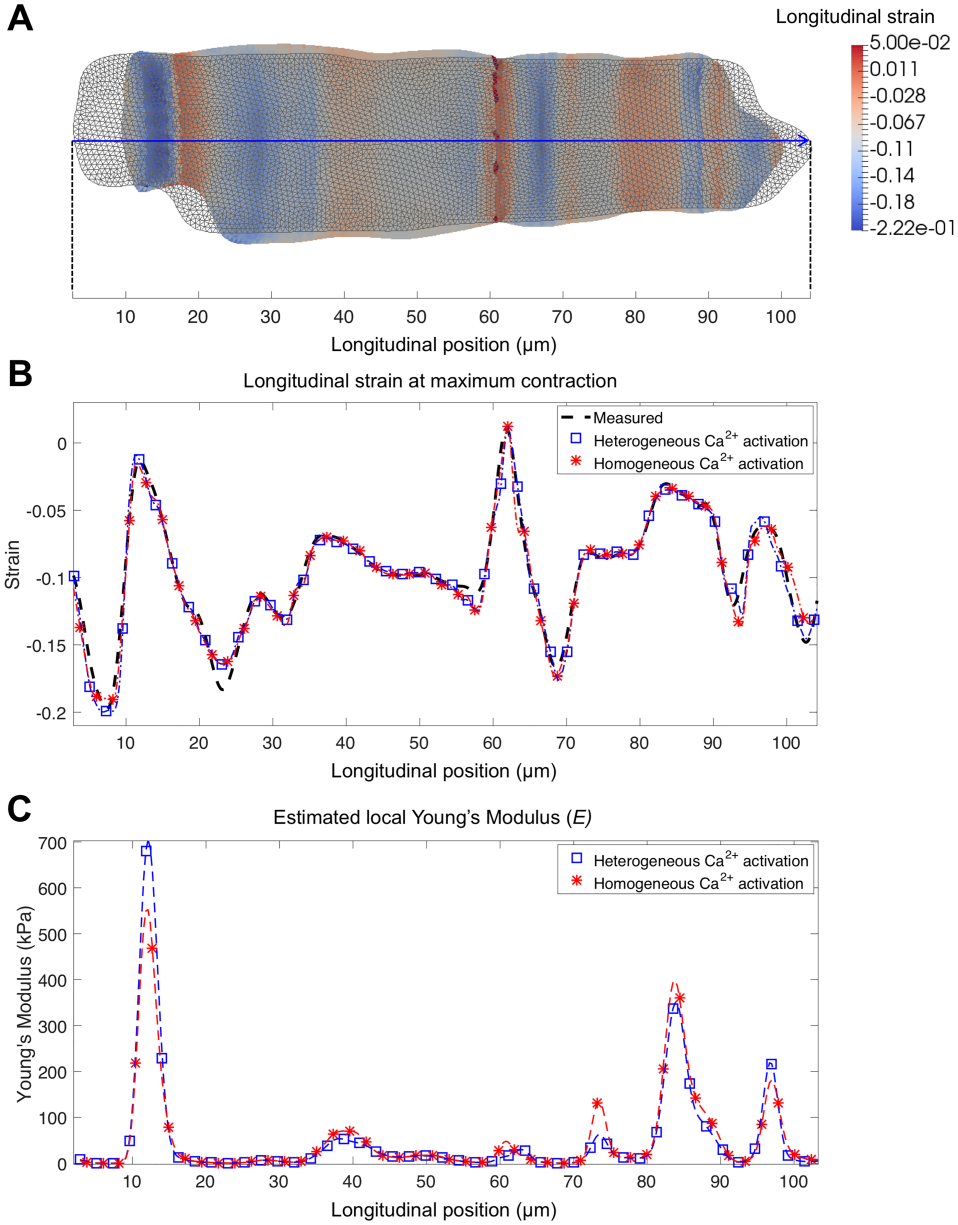
Cell-specific simulation results after solving the inverse problem. A: Undeformed (grey) and deformed cell mesh in the maximum contraction time frame. Colormap indicates the simulated longitudinal strain. B: Measured (black dashed line), and simulated strain curves obtained along the line-scan (blue arrow) at maximum contraction time frame, for a homogeneous (red line) and heterogeneous (blue line) activation. C: Estimated Young’s modulus (*E*) for a homogeneous (red line) and heterogeneous (blue line) activation.

The cell-specific electromechanical simulations were successfully performed for three different cardiomyocytes, both with homogeneous and heterogeneous [*Ca*^2+^]—activation conditions. The cell meshes contained on average 9680 elements and 5000 nodes. The RMSE of the cell-specific fittings of the local longitudinal strain was below 0.01 in all cases, with either homogeneous or heterogeneous activation, as detailed in Table 2. Fig 5A shows the 2D cell mesh (grid) corresponding to the cardiomyocyte illustrated in Fig 1A together with the deformed mesh at maximum contraction time (colormap mesh showing simulated local longitudinal strain), for the heterogeneous activation. Fig 5B shows the manually measured longitudinal strain along the line-scan (dash black), together with the simulated strain at the maximum contraction time frame with the optimised *E*_*i*_ for the case of homogeneous (red line) and of heterogeneous (blue line) [*Ca*^2+^]—activation. The figure demonstrates the heterogeneity in the longitudinal strain during deformation. In addition, the two simulated strain curves were quite similar compared to the experimentally measured strain. The local estimated Young’s moduli along the line-scan is plotted for both simulated scenarios in Fig 5C, showing almost identical results in both cases, except at the location where the maximum Young modulus is obtained. A similar behaviour was obtained for the other two cells.

**Table 2:** Results of the cell-specific simulations performed for three different cardiomyocytes, for both [*Ca*^2+^]-activation (Act.) scenarios: Homogeneous (homo.) and heterogeneous (hetero.)

Cell	Act.	RMSE	Shortening	$\bar{E}$ (kPa)	*f*_*max*_ (kPa)	*α* (kPa)	*ρ*
**N° 1**	Homo.	0.0032	8.2%	44.67	54.33	0.042	0.75**
	Hetero.	0.0042	8.1%	49.38	54.33	0.035	0.73**
**N° 2**	Homo.	0.0071	10.4%	59.59	62.47	0.035	0.66**
	Hetero.	0.0077	10.3%	58.50	54.33	0.028	0.61**
**N° 3**	Homo.	0.0039	9.3%	21.58	66.82	0.017	0.73**
	Hetero.	0.0052	9.5%	25.65	70.63	0.015	0.73**

*ρ* denotes Pearson’s correlation coefficient;

** *p* < 0.001.

A summary of the results from the optimisation procedure (the final RMSE reached, the cell global shortening, mean Young’s moduli $\bar{E}$, maximum force *f*_*max*_ and the elastic parameter of Robin boundary condition *α*) for the three simulated cells is given in Table 2. The Pearson’s correlation, *ρ*, between the local deformation and estimated *E* is also reported in Table 2. In all simulations, a significant positive correlation between *E* and strain was obtained. It can be noticed that the correlation obtained in the numerical simulations (*ρ* = 0.7 in average) was very similar to the correlation coefficient obtained from the sensitivity analysis (*ρ* = 0.73).

## Discussion

We have developed a two-dimensional electromechanical model of a cardiomyocyte that takes into account the heterogeneity in the mechanical properties of a cardiac cell, overcoming the limitations from previous studies, which assumed an uniform distribution of mechanical properties throughout the cell [23–25, 29, 30]. Our proposed two-dimensional electromechanical cell model allows for taking into account local material parameters that guide the electromechanical behaviour of the cell. These parameters are estimated by using the experimentally measured local deformation and cytosolic [*Ca*^2+^] concentration of the cell during contraction in an inverse problem procedure. The correlation between the amplitude and duration of cell contraction and the different mechanical properties was assessed by means of a sensitivity analysis. Finally, the cell-specific variation of the Young’s modulus along the longitudinal axis of the isolated cell was successfully estimated in three different cardiac cells by means of an inverse problem procedure, showing that a heterogeneous distribution of the Elastic Modulus within the cell was able to explain the contraction pattern exhibited by the cardiomyocyte in three different cells.

The results from the sensitivity analysis have shown that the duration of the cell contraction was mainly determined by the duration of the [*Ca*^2+^] transient. This result is also supported by previously published studies on cardiomyocytes reporting a significant correlation between both local and global time of sarcomere re-lengthening (*τ*_*sl*_) and the time of [*Ca*^2+^] decay (*τ*_*fall*_) [4]. In loaded muscle, force regulates myofilament *Ca*^2+^ sensitivity via *TnC*, on this will have an effect on the decline of the free *Ca*^2+^. However, in our case, we have freely contracting cells, and therefore no force develops and thus decline of *Ca*^2+^ will be mainly governed by SERCA. Results of the sensitivity analysis also suggested that regional differences in the cellular deformation were mainly due to the local elasticity of the cell rather than the local cytosolic [*Ca*^2+^]. The results from the cell-specific simulations also support this hypothesis, since local cell elasticity estimated when considering an homogeneous [*Ca*^2+^] activation function was practically identical to the local elasticity estimated when the local [*Ca*^2+^] transients were used to construct the activation functions. This suggests that the regional variation of the cell’s elasticity is orders of magnitude higher than changes induced by the [*Ca*^2+^] heterogeneity, and therefore a global homogeneous activation could be considered instead. Also, the averaged Young’s moduli identified in our simulations and the cell’s global shortening were in close agreement with reported values for rat cardiac cells [11, 13, 25].

Previous studies have demonstrated the significant heterogeneity of the Young’s modulus in the cardiac cell [11–14]. The study by Shroff et al. [15] stated that the stress fibres shown areas with high stiffness (elastic modulus between 100–200 kPa) embedded in softer parts of the cell (elastic modulus between 5–30 kPa). An analysis of fluorescent images together with the elasticity maps obtained by AFM revealed that the variation of elastic properties across the cardiomyocyte was correlated with the cytoskeleton heterogeneity [41], specifically to the actin network of the cell cytoskeleton [11]. Additionally, changes in mechanical properties and cytoskeleton reorganisation have been also described in cell aging [13] and under pathogenesis [11]. Regarding the sarcomere uniformity inside the cell, Brady [42] reported that in isolated unattached cardiac cells, the sarcomere pattern both at rest and during isotonic contractions was remarkably uniform, resulting in an overall sarcomere length variation of only ±6%, and the major sarcomere length deviation was noted near the nuclei. However, regional variations in the onset of relaxation were observed during the active shortening that lead to a greater dispersion of sarcomere lengths. All theses experimental studies support our results showing that the heterogeneity in the cellular deformation seems to be mainly caused by the regional differences in cell elasticity and that, this variation can be explained by differences in the cytoskeleton architecture and organelles.

Some comments regarding the limitations of the present model are in order. First, a quite simple cell geometry has been assumed, considering plane stress, and assuming that the out-of-plane deformation is such that the volume of the cell is preserved. However, since the data was available only in one-dimension (longitudinal deformation), it is believed that a 2D model is accurate enough for reproducing the experimental data. For this reason (lack of experimental data on the transversal direction), only heterogeneity in the longitudinal direction of the cell was considered while homogeneity in the transversal direction was assumed. Secondly, the viscous component of the cardiomyocyte and the effect of velocity on contraction were not modelled, and therefore the dependency on the frequency of the [*Ca*^2+^] signal was not considered and the variation of Young Modulus through the cell beat (dynamic Young Modulus) neither. Nevertheless, this was out of the scope of the study since we were only interested in the estimation of the static Young’s modulus. The tension dependence *Ca*^2+^ binding to *TnC* was indirectly modelled through the function *f*_*TCa*_(*t*). In this study, due to the lack of force measurements, it was fitted to the normalised cell deformation to catch the time course of *Ca*^2+^ binding to and dissociation from *TnC*. However, if force measurements were available *f*_*TCa*_(*t*) can be fitted to the force transient and therefore modelling the dependence *Ca*^2+^ binding to *TnC*. We did not include a detailed model for *Ca*^2+^ dynamics and the associated propagating calcium waves. We acknowledge that these kind of models can provide more information about the factors that can lead to heterogeneities in the *Ca*^2+^ transient. However, our model considered the local experimentally measured *Ca*^2+^ transients instead, which allow us to parametrise the model for each specific cell. Regarding the *Ca*^2+^ wave propagation, healthy rat ventricular cells have a very high dense T-Tubule network, thus, in a freely contracting cell, the *Ca*^2+^ release is taking place almost at the same time along the cell. This can be appreciated in the line-scan image corresponding to cytosolic [*Ca*^2+^] (Fig 1D), where no delay in the *Ca*^2+^ release along the longitudinal axis of the cell can be appreciated. Therefore, we assumed no *Ca*^2+^ wave propagation in our model. Also structural heterogeneity in contractile elements might be present and activation-contraction coupling can vary locally. While we have not explicitly included this in the model, we think that this is (at least partially) captured in our active contraction model by the term *A* (Eq 3) describing the local uptake of *Ca*^2+^. A more detailed model of activation-contraction coupling is out of the scope of this paper. Even by simplifying most of the biochemical and biomechanical processes, the present model was able to successfully recreate the local and global behaviour of electrically stimulated contracting cardiomyocytes. Thirdly, the manual measurements of the experimental data are prone to some errors that can affect the final results. We acknowledge that in order to further validate our model, experimentally measured elastic modulus should be compared with model estimations in the same cell. Further experiments like the one proposed by Sugiura et el [43] which allow the measurement of passive forces together with local variations in sarcomere length, could provide data for an experimental validation of the estimated material parameters. Finally, we have just tested our model in healthy cells, where *Ca*^2+^ peak heterogeneities are not higher than around 15–20%. However, it would be interesting to model cells that present calcium handling abnormalities as, for instance, subcellular *Ca*^2+^ discordant alternans, where the local changes in the *Ca*^2+^ peak may also be higher in different parts of the cell.

## Conclusion

In conclusion, our proposed electromechanical model of a cardiomyocyte seems to be a good alternative to assess the local mechanical properties such as passive elasticity within a normal cardiomyocyte and offers a relevant basis for understanding the spatial heterogeneity in the mechanical properties and deformation. Our results suggested that the heterogeneity in the local deformation is mainly caused by the regional variation on the cell elasticity that could be attributed to local changes in the cytoskeleton of the cell and the different organelles. Therefore, this model could provide a starting point for an extended analysis of the relationship among regional cell mechanical behavior and the underlying biochemical and/or structural environment, in both physiological and pathological contexts.

## Supporting information

S1 Appendix Supplementary methods. (PDF)

S1 Fig Fitting SL-active tension relationship. Fitting of the experimental data obtained by Weiwad et al [33], highlighting the SL-active tension relationship for skinned cardiac cells with the expression *T*_*act*_ given by equation S1.22 in S1 Appendix. Data points series correspond to pCa values of 5.7, 5.46 and 4.9. Solid lines indicate the best solution obtained when fitting simultaneously all these data.
(TIFF)
