# Supplementary material for: A two dimensional electromechanical model of a cardiomyocyte to assess intra-cellular regional mechanical heterogeneities
Source: PLoS One. 2017 Aug 24;12(8):e0182915. doi: 10.1371/journal.pone.0182915 (PMC5570434; doi:10.1371/journal.pone.0182915)
Supplement: S1 Appendix — (PDF) [file pone.0182915.s001.pdf]

S1 Appendix. Supplementary Methods

1. Description of the model

Passive stress

The strain-energy function of a nearly-incompressible Mooney Rivlin material is as follows:

$$W = C_{10}(\bar{I}_1 - 3) + C_{01}(\bar{I}_2 - 3) + D_1(J-1)^2 \quad \text{(S1.1)}$$

The second Piola-Kirchhoff stress tensor is calculated from the strain energy function as follows:

$$S = 2\frac{dW(\bar{I}_1,\bar{I}_2,J)}{dC} \quad \text{(S1.2)}$$

Applying the chain rule:

$$S = 2\frac{dW(\bar{I}_1,\bar{I}_2,J)}{dC} = 2\left(\frac{dW}{d\bar{I}_1}\frac{d\bar{I}_1}{dC} + \frac{dW}{d\bar{I}_2}\frac{d\bar{I}_2}{dC} + \frac{dW}{dJ}\frac{dJ}{dC}\right) \quad \text{(S1.3)}$$

We also know that the modified invariants are defined as:

$$\bar{I}_1 = J^{-2/3} I_1 \quad \text{(S1.4)}$$

$$\bar{I}_2 = J^{-4/3} I_2 \quad \text{(S1.5)}$$

so, if we calculate the following derivatives:

$$\frac{dJ}{dC} = \frac{J}{2}C^{-1} \quad \text{(S1.6)}$$

$$\frac{d\bar{I}_1}{dC} = \frac{d\left(J^{-2/3}I_1\right)}{dC} = \frac{dJ^{-2/3}}{dC}I_1 + J^{-2/3}\frac{dI_1}{dC} \quad \text{(S1.7)}$$

knowing that $\frac{dI_1}{dC} = \mathrm{I}$, $\frac{dJ^{-2/3}}{dC} = -\frac{1}{3}J^{-2/3}C^{-1}$ and $I_1 = tr\left(C\right)$ the final expression of $\frac{d\bar{I}_1}{dC}$ is:

$$\frac{d\bar{I}_1}{dC} = -\frac{1}{3}J^{-2/3}C^{-1}I_1 + J^{-2/3}\mathrm{I} = -\frac{1}{3}J^{-2/3}C^{-1}tr\left(C\right) + J^{-2/3}\mathrm{I} = J^{-2/3}\left(\mathrm{I} - \frac{1}{3}tr\left(C\right)C^{-1}\right) \quad \text{(S1.8)}$$

The derivative of the modified second invariant is as follows:

$$\frac{d\bar{I}_2}{dC} = \frac{d\left(J^{-4/3}I_2\right)}{dC} = \frac{dJ^{-4/3}}{dC}I_2 + J^{-4/3}\frac{dI_2}{dC} \quad \text{(S1.9)}$$

knowing that $\frac{dI_2}{dC} = I_1\mathrm{I} - C$ and that $\frac{dJ^{-4/3}}{dC} = \frac{-2}{3}J^{-4/3}C^{-1}$, the final expression of $\frac{d\bar{I}_2}{dC}$ is:

$$\frac{d\bar{I}_2}{dC} = -\frac{2}{3}J^{-4/3}C^{-1}I_2 + J^{-4/3}\left(I_1\mathrm{I} - C\right) = J^{-4/3}\left(I_1\mathrm{I} - C - \frac{2}{3}C^{-1}I_2\right) \quad \text{(S1.10)}$$

and by substituting the expression of the 1st and 2nd invariants: $I_1 = tr(C)$ and $I_2 = \frac{1}{2}\left(\left(tr(C)\right)^2 - tr\left(C^2\right)\right)$ we have:

$$\frac{d\bar{I}_2}{dC} = J^{-4/3}\left(tr(C)\mathrm{I} - C - \frac{2}{6}\left[\left(tr(C)\right)^2 - tr\left(C^2\right)\right]C^{-1}\right) \tag{S1.11}$$

Finally, the derivate of the strain-energy function with respect to the modified invariants and J are:

$$\frac{dW}{d\bar{I}_1} = C_{10} \tag{S1.12}$$

$$\frac{dW}{d\bar{I}_2} = C_{10} \tag{S1.13}$$

$$\frac{dW}{dJ} = 2D_1(J-1) \tag{S1.14}$$

Finally, substituting equations 1.8, 1.11, 1.12, 1.12 and 1.14 in equation 1.3, the expression of the second Piola-Kirchhoff stress tensor takes on the form:

$$S = 2\left(C_{10}J^{-2/3}\left(\mathrm{I} - \frac{1}{3}tr(C)C^{-1}\right) + C_{01}J^{-4/3}\left(tr(C)\mathrm{I} - C - \frac{2}{6}\left[\left(tr(C)\right)^2 - tr\left(C^2\right)\right]C^{-1}\right) + D_1(J-1)JC^{-1}\right)$$

(S1.15)

We can separate the stress expression in deviatoric (S_{dev}) and volumetric (S_{vol}) terms as $S = S_{dev} + S_{vol}$:

$$S_{dev} = 2\left(C_{10}J^{-2/3}\left(\mathrm{I} - \frac{1}{3}tr(C)C^{-1}\right) + C_{01}J^{-4/3}\left(tr(C)\mathrm{I} - C - \frac{2}{6}\left[\left(tr(C)\right)^2 - tr\left(C^2\right)\right]C^{-1}\right)\right) \tag{S1.16}$$

$$S_{vol} = 2D_1(J-1)JC^{-1} \tag{S1.17}$$

S_{dev} corresponds to the expression of passive stress $S_{pas.}$

For consistency with linear elasticity in the limit of small strains, it is necessary that:

$$\mu = 2\left(C_{10} + C_{01}\right) \tag{S1.18}$$

and

$$K = 2D_1 \tag{S1.19}$$

where K is the bulk modulus and μ is the shear modulus. We can relate K and μ with the Young's Modulus, E, and Poisson's ratio, v, through the following relationships:

$$E = \frac{9K\mu}{3K + \mu} \tag{S1.20}$$

$$\nu = \frac{3K - 2\mu}{2(3K + G)} \tag{S1.21}$$

As the cell was modelled as a nearly-incompressible material Poisson's ratio was set to 0.49. We have defined C_{01}=C_{10}/50.

Active stress

The active stress generated by the active fibres within the cell was modelled as an active tension, T_{act}, generated along the direction given by the sarcomere orientation in the reference configuration, d^k, calculated as: $S_{act} = T_{act}D^k$, where the tensor $D^k = d^k \otimes d^k$ is defined by the fibre system direction vector d^k. Hence, an anisotropic active contraction of the cardiomyocyte is assumed. In this regard, only fibers in the longitudinal direction were considered so $k = 1$. The active tension, T_{act}, was computed as:

$$T_{act} = A \cdot f_{max} \cdot exp\left\{-\left(\frac{\varepsilon - \varepsilon_{opt}}{s}\right)^2\right\} \tag{S1.22}$$

where $\varepsilon = \frac{1}{2}\left(\bar{C} : D^k - 1\right)$ is the Green strain along the fiber direction d^k, ε_{opt} is the optimal deformation at the maximal activation state, and s represents the sensitivity to the actin-myosin overlap. The parameter f_{max} is the maximal tension that can be delivered by the sarcomere. Parameters ε_{opt}, s and f_{max} were identified from experimental data acquired on skinned rat cardiac myocytes (Weiwad et al 2010).

The overall set of data points reported by Weiwad et al. was fitted for pCa (pCa = -log[Ca2+]) ranging from 4.9 to 5.7 with the expression of actives stress Tact (Eq. S1.22), for a resting sarcomere length $SL_0 = 1.9\ um$ and a Hill exponent $nH = 2.6$, in agreement with the experimental data. The values identified after the non-linear regression procedure were f_{max} = 54.33, ε_{opt} = 0.23 *and* s = 0.24. Figure S1 shows the results of the fitting of Eq. S1.22 to the experimental SL-active tension data points at different Ca2+ activation levels.

A was defined as an activation [Ca^{2+}]-dependent function modelling the excitation-contraction coupling. This process was described as follows:

$$A(t) = \frac{Z_{max}^{nH}}{Z_{50}^{nH} + Z_{max}^{nH}} \frac{Z(t)}{Z_{max}} f_{TCa}(t) \tag{S1.23}$$

The first term of the equation corresponds to a Hill-type sigmoid function, where nH is the Hill coefficient ($nH > 0.0$), Z_{max} represents the maximum peak of cytosolic [Ca^{2+}] and Z_{50} the half-maximum concentration of cytosolic [Ca^{2+}]. The second term, $Z(t)/Z_{max}$,

corresponds to the normalized [Ca2+] transient. *Z(t)* was approximated as a sum of two exponential functions as follows:

$$Z(t) = a\left(e^{-kfall \cdot t} - e^{-krise \cdot t}\right) + b \qquad \text{(S1.24)}$$

where a and b define the amplitude and the baseline of the [Ca^{2+}] transient and k_{fall} and k_{rise} the rates constants of [Ca^{2+}] decay and rise respectively. Therefore, the corresponding time constants were calculated as $\tau_{fall} = 1/\ k_{fall}$ and $\tau_{rise} = 1/\ k_{rise}$. The transient maximum amplitude, Z_{max} corresponds to the time point where the 1st derivative of Z(t) is 0. Therefore, Z_{max} can be calculated as follows:

$$\frac{dZ(t)}{dt} = A\left(k_{rise}e^{-k_{rise} \cdot t} - k_{fall}e^{-k_{fall} \cdot t}\right) = 0 \rightarrow t = \frac{log\left(k_{fall}/k_{rise}\right)}{k_{fall} - k_{rise}} \qquad \text{(S1.25)}$$

And substituting Eq 1.25 in Eq. 1.24 we find the value of Z_{max} as follows:

$$Z_{max} = A\left(\frac{1}{\left(k_{fall}/k_{rise}\right)^{\frac{k_{fall}}{k_{fall}-k_{rise}}}} - \frac{1}{\left(k_{fall}/k_{rise}\right)^{\frac{k_{rise}}{k_{fall}-k_{rise}}}}\right) \qquad \text{(S1.26)}$$

The last term in equation Eq. 1.23, $f_{TCa}(t)$, is a unitary function that describes the temporal behaviour of the [Ca^{2+}] binding to and dissociation from troponin (TnC) in the myofilaments, and therefore the [Ca^{2+}] regulation of contraction. It was defined as the multiplication of two exponentials as follows:

$$f_{TCa}(t) = \frac{\left(1 - e^{-(t/\tau_c)^{\beta}}\right)\left(e^{-((t-t_b)/\tau_r)^{\beta}}\right)}{\left(1 - e^{-(t_p/\tau_c)^{\beta}}\right)\left(e^{-((t_p-t_b)/\tau_r)^{\beta}}\right)} \qquad \text{(S1.27)}$$

where τ_c and τ_r are the contraction and relaxation time constants respectively, β is an exponent between 1 and 2, and t_p corresponds to the time to peak, such that maximum of $f_{TCa}(t)$ is 1.

Total Stress

The total stress is the sum of the effective stress, calculated as the addition of passive and active stresses $S_{pas} + S_{act}$, and the volumetric stress S_{vol}:

$$S = S_{pas} + S_{act} + S_{vol} = S^{eff} + 2D_1\left(J-1\right)JC^{-1} = S^{eff} + K\left(J-1\right)JC^{-1} \qquad \text{(S1.28)}$$

Therefore, the force equilibrium equation: in weak formulation is defined as:

$$\int_{\Omega^{(0)}} S\left(\underline{u}\right) \cdot \partial\varepsilon\left(\underline{v}\right)dV = \int_{\Omega^{(0)}} \left(S^{eff}\left(\underline{u}\right) + K(J-1)JC^{-1}\right) : \partial\varepsilon\left(\underline{v}\right)dV = 0, \forall \underline{v} \qquad \text{(S1.29)}$$

where ε is the Green strain tensor, Ω is the initial (undeformed configuration), $\underline{u}$ is the displacement vector and $\underline{v}$ is a vector test function.

2. Finite-element simulation

The equilibrium equation was numerically solved using the finite element method with the software *Sfep*y. A non-linear finite-element model was used, following the total Lagrangian approach, where the discrete equations are formulated with respect to the reference configuration. 2D meshes with triangular elements were constructed to describe the geometry of the cardiac cells. The Newton solver with backtracking line-search from *Sfepy* was used. This system is solved iteratively by the Newton Method until a convergence criterion is met. The solver's parameters we defined were as follows:

- 'i_max' : 7, (The maximum number of iterations.)
- 'eps_a' : 1e-6, (The absolute tolerance for the residual, i.e. $||f(x^i)||$.
- 'eps_r' : 1.0, (The relative tolerance for the residual, i.e. $||f(x^i)|| / ||f(x^0)||$.
- 'macheps' : 1e-16, (The float considered to be machine "zero".)
- 'lin_red' : 1e-2, (The linear system solution error should be smaller than (eps_a * lin_red).
- 'ls_red' : 0.8, (The step reduction factor in case of correct residual assembling).
- 'ls_red_warp' : 0.1, (The step reduction factor in case of failed residual assembling (e.g. the "warp violation" error caused by a negative volume element resulting from too large deformations).
- 'ls_on' : 1.1, (Start the backtracking line-search by reducing the step, if $||f(x^i)|| / ||f(x^{i-1})||$ is larger than ls_on.)
- 'ls_min' : 1e-5, (The minimum step reduction factor.)

The solution is considered converged if in maximum 'i_max' iterations, the absolute residual $||f(x^i)||$ is < 'eps_a' and the relative residual, $||f(x^i)|| / ||f(x^0)||$ is < 'eps_r'.

For the time solver, a dt=0.005s was set.

3. Inverse problem procedure

The local elastic Young's moduli (E_i) along the longitudinal axis of the cell were estimated automatically by a two-step constrained non-linear optimisation algorithm implemented in Matlab. Specifically, we used the function "*fmincon*" with the Sequential Quadratic Programming (SQP) method. The vector of parameters to estimate include all the local E_i, f_{max} / f^0_{max} and α/α^0, being f^0_{max} = 54.33 kPa, and α^0 = 0.02 kPa.

We scaled all the parameters of the vector to be within the same order of magnitude by taking its logarithm in order to have the same sensitivity in all the parameters in the minimisation algorithm. We defined upper and lower bounds for the parameter vector. The lower bounds that were defined for E_i, f_{max} and α were: [-Inf, log(0.5) log(0.1)] respectively, and the upper bound were: [log(1000), log(5), log(100)].

The optimisation algorithm consists of minimising an objective function, Φ, defined as the root mean square error (RMSE) between the simulated and original experimental strain profiles along the longitudinal axis of the cell at the maximum contraction time frame. Hence, the estimation problem consists in searching the parameter set, θ, that minimises Φ starting from an initial parameter set ($\theta^0 = E_i^0$), until the stopping criterion $\left\|\theta^n - \theta^{n+1}\right\| < 10^{-6}$ on Φ is reached. In order to avoid local minimum solutions, we repeated the procedure several times with different θ^0 keeping the parameter set with a minimum value of Φ as the final solution of the inverse problem.

We propose to estimate the local E_i in two steps. First, a less refined estimation was performed by diving the cell in approximately 20 regions along the cell's longitudinal axes, and assuming a piecewise linear variation of the logarithm of the elastic Young's modulus along the longitudinal axis of the cell as described in Eq. 8. After the first minimisation process is completed, a second optimisation step is performed in which, the cardiomyocyte was divided in approximately 200 segments along the longitudinal direction, where the local Young's modulus was determined. The Young moduli identified in the first step were interpolated into the new segments and used as initial seeds for the second optimisation algorithm.
