# Supplementary figures and images for: A two dimensional electromechanical model of a cardiomyocyte to assess intra-cellular regional mechanical heterogeneities
Source: PLoS One. 2017 Aug 24;12(8):e0182915. doi: 10.1371/journal.pone.0182915 (PMC5570434; doi:10.1371/journal.pone.0182915)

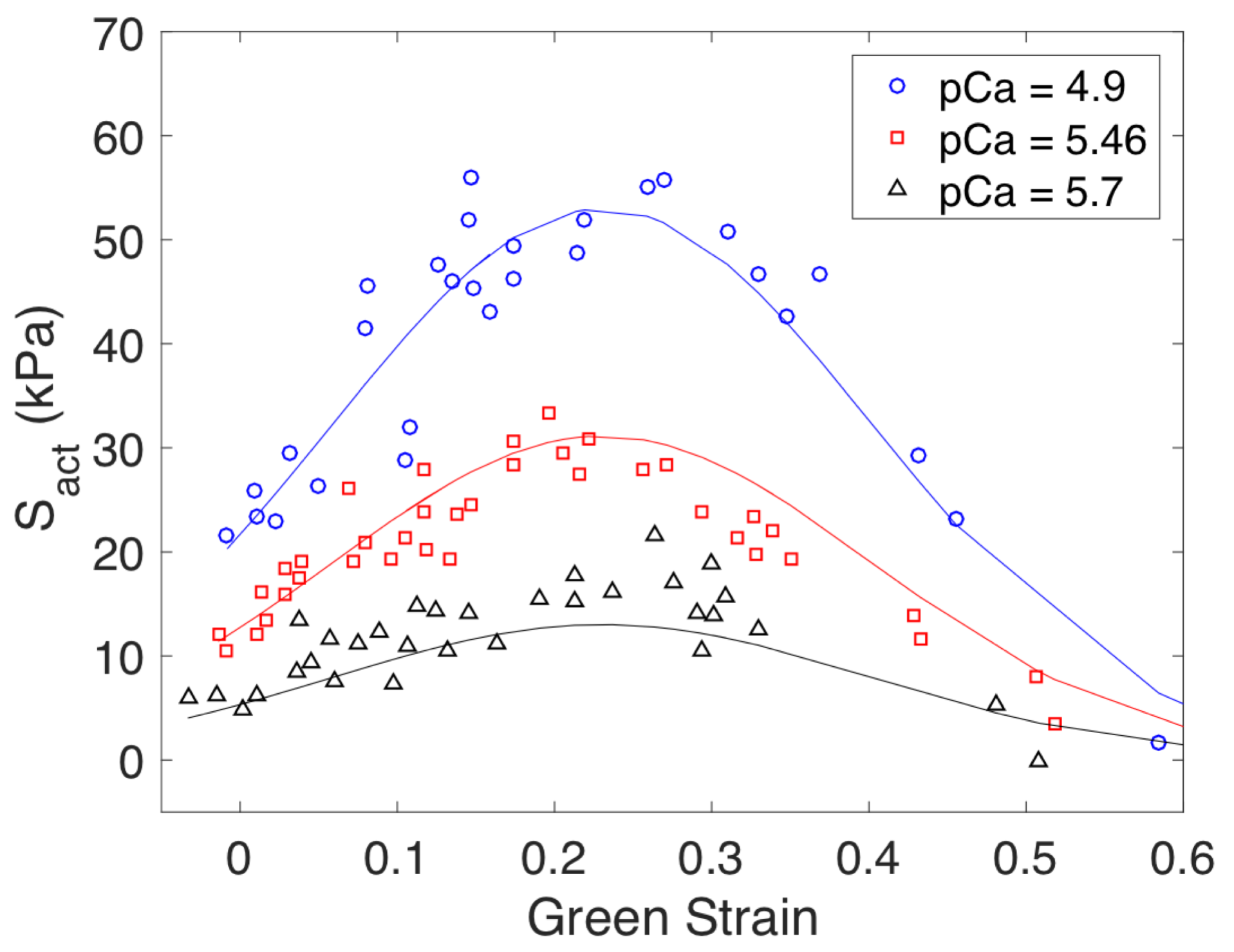

Supplement: S1 Fig — Fitting of the experimental data obtained by Weiwad et al [33], highlighting the SL-active tension relationship for skinned cardiac cells with the expression Tact given by equation S1.22 in S1 Appendix. Data points series correspond to pCa values of 5.7, 5.46 and 4.9. Solid lines indicate the best solution obtained when fitting simultaneously all these data. (TIFF) [file pone.0182915.s002.tiff]
